# Ethicality forms a challenge in the implementation of structured violence risk screening: a qualitative study of an intervention in two Swedish emergency psychiatric units

**DOI:** 10.3389/fpsyt.2026.1849481

**Published:** 2026-06-24

**Authors:** Rose-Marie Lindkvist, Anders Håkansson, Lars Garpenhag

**Affiliations:** 1 Department of Clinical Sciences Malmö, Psychiatry, Lund University, Lund, Sweden; 2 Department of Clinical Sciences Lund, Psychiatry, Lund University, Lund, Sweden; 3 Malmö Addiction Center, Region Skåne, Malmö, Sweden

**Keywords:** clinical ethics, psychiatric emergency services, qualitative research, risk management, violence

## Abstract

**Introduction:**

Violence is common in psychiatric settings. Routine violence risk screening in acute psychiatric settings may be used to identify needs for preventive measures. However, introducing general screening for violence risk in acute psychiatric care may evoke ethical controversy among professionals which in turn will influence implementation. This study aimed to assess professionals’ ethical acceptability of an intervention introducing routine use of Violence risk screening-10 (V-RISK-10) in two psychiatric emergency units in Malmö, Sweden.

**Methods:**

Semi-structured interviews with developers and staff (n=6) based on the theoretical framework of acceptability were analyzed using content analysis, with focus on ethics and ethicality.

**Results:**

Although respondents were positive towards the use of V-RISK-10, in terms of its potential to promote uniform and fair assessments, they also expressed concerns. Respondents described ethical risks, tied to the accuracy and interpretation of results and their operationalization as an element in risk management. The perceived ethical acceptability of the intervention rested on a balance between different individual and collective interests.

**Discussion:**

Based on authentic clinical realities findings indicate an ethical complexity of routine violence risk screening. In the studied local implementation context, violence risk screening may fit with the ethical values of psychiatric staff in an emergency care setting. Care needs to be taken in the implementation process to ensure that necessary knowledge is disseminated throughout the healthcare organization, and clear guidelines are in place for the use of screening results.

## Introduction

Violence is a common problem in healthcare. In surveys, significant proportions of Swedish health professionals have indicated that they have been subjected to threats or violence (1, 2). Aside from causing physical injuries, patient violence and threats have detrimental psychological, emotional and social consequences for both the victims (3–7) and the wider treatment environments in which they take place (8, 9). Perpetrators themselves risk not only to unwilfully hurt others, but also to become subject to coercive measures and criminal prosecution. Psychiatric settings belong to the ones most exposed to aggressive behavior displayed by patients (2, 10, 11). In acute psychiatric settings, typically characterized by quick decision-making and limited predictive information, prevention of violence may be particularly challenging (12).

Efforts to prevent psychiatric inpatient violence have brought risk assessment tools to the forefront. Numerous clinical instruments – assessing both short- and long-term risks – have been developed, validated and used in clinical practice (13). Sound psychometric properties, however, do not in themselves guarantee the successful use of an instrument in real life situations. Contextual factors, and dimensions such as feasibility and acceptability, shape the chances of a complex healthcare intervention to become a durable routine (14–16). As noted by Chaimowitz and colleagues (17), the implementation of instruments for violence risk assessment in specific clinical settings remains only scarcely explored.

Acceptability of healthcare interventions, meaning the extent to which those involved in delivering or receiving care consider an intervention to be appropriate, is crucial, as this will shape implementation, and potentially modify effectiveness (18). While the acceptability of violence risk screening in different psychiatric settings has been examined, the literature on staff perceived acceptability has been limited by an oftentimes narrow focus on the practical utility of instruments (9, 20–22). This has left us with a limited understanding of other factors that make violence screening appear more or less acceptable to healthcare professionals. Low predictive accuracy and its consequences for clinical decision making and resource allocation (23–26), risks associated with stigma (27), and the potential of systemic bias towards already marginalized groups (28), are all examples of ethically pertinent issues that have been observed in the literature. However, although staff concerns regarding certain ethical aspects of violence risk assessment in psychiatry have been touched upon previously (29–31) we know little about how the fit between such interventions and staff members’ ethical values influence the implementation of structured violence risk assessment in clinical practice.

In 2023 an intervention based on routine use of the instrument Violence risk screening-10 (V-RISK-10) (32) to screen newly admitted patients was implemented in two psychiatric emergency units in Malmö, Sweden. The aim of this study was to assess the ethical acceptability of the intervention as perceived by staff members, as well as how this dimension was considered during the development phase.

## Materials and methods

### The intervention and setting

The intervention was implemented at two psychiatric emergency units in a university hospital in Malmö, Sweden, one general psychiatric and one specialized in addiction, with a clinical staff (physicians, nurses and nursing assistants) of approximately 30 and 40 respectively. The general psychiatric emergency unit handled acute psychoses and observation of patients with elevated suicide risk, severe self-harm, violent behaviour or eating disorders, and functioned as acute admission unit for a total of six inpatient wards and one day-patient care ward. The addiction emergency unit handled acute intoxication needs and observation of patients with substance use disorders, and formed the acute admission unit for three wards, one of which was part of the emergency unit itself. Thus, it provided both emergency triage and care. The natural catchment area, including the city of Malmö and suburban and rural areas in its vicinity, has a population of approximately 550,000–650,000.

The intervention consisted of two main elements: (i) training to raise staff awareness of violence risk and prepare them for the use of V-RISK-10 for violence risk screening, and (ii) the integration of V-RISK-10 into the admission procedure. V-RISK-10 is a brief instrument for the assessment of short-term violence risk, specifically developed to assess acute psychiatric patients (32, 33), based on a structured professional judgment approach (34).

The training (i) consisted in current staff – including temporary members – e.g. medical residents – taking part of a training package (one online lecture and two half-day training sessions) aimed at raising awareness about violence risks and training them in the conduction of screenings using V-RISK-10 and management of risks. Late comers were offered a compressed training package, consisting of the filmed online lecture and one half-day training session.

The implemented routine for violence screening (ii) consisted in the conduction and documentation of screenings using V-RISK-10 at admission of patients. Results were supposed to form the basis for decisions on the need for further assessment of the risk of inpatient violence (using Brøset Violence Checklist, 35). Screening results would also serve as a guideline for preventive measures aiming at reducing immediate risk during inpatient care, such as adapting care, approach and environment, and informing colleagues. In addition, screening was supposed to enhance long-term outcomes through work with vulnerability as well as protective factors in the individual patient. The emphasis on risk management routines was greater in the addiction unit, which was integrated with an inpatient ward, than in the psychiatric unit. The integration of V-RISK-10 into the admission routines was overseen by a volunteer, mixed-profession group at each unit.

### Data collection

Data were collected in semi-structural individual interviews with persons involved in the development of the intervention (n=2), and with staff members in the two units where the intervention was implemented (n=4). The intervention was implemented during 2023 and interviews were conducted in 2024 and 2025.

Recruitment of staff members took place through e-mails from the project leader of the implementation to members of the staff in the two units, as well as physical advertising in the workplace. Reminders were made, both via e-mail and at staff meetings, and targeted invitations were sent out to certain categories of staff (unit managers and peer support staff). Developers were invited by e-mail from the interviewers. Interviews were conducted either in respondents’ workplaces or using video conference technology (Zoom). Interviews (lasting 20–57 minutes) were carried out by the first and last authors who had backgrounds in qualitative research, including interviews with healthcare professionals in psychiatry. The interviewers had no professional relationship with the study participants and were not involved in development or implementation of the studied intervention. AH (author 2) was involved in development and implementation as a member of the steering committee and in study design but not involved in data collection or analysis.

The interviews were conducted using two different interview guides with open-ended questions, one for developers and one for staff. Both were developed based on the theoretical framework of acceptability (TFA) by Sekhon and colleagues (18). Based on an understanding of acceptability as “[a] multi-faceted construct that reflects the extent to which people delivering or receiving a healthcare intervention consider it to be appropriate, based on anticipated or experienced cognitive and emotional responses to the intervention” (18, p. 4) the TFA identifies seven component constructs: affective attitude, burden, ethicality, intervention coherence, opportunity costs, perceived effectiveness and self-efficacy (18). All interviews began with an open question asking participants to speak generally about their experiences of their work related to the screening tool. Additional questions were phrased to cover each component construct of acceptability according to Sekhon and colleagues (18). To cover the construct ‘ethicality’ we developed open questions such as these directed to staff: How do you view the ethical aspects of screening? Could screening be considered right or wrong, fair or unfair? If so, in what situations? The corresponding questions to developers were similar but adapted to their specific role: How did you in the developing group consider the ethical aspects of screening for the risk of violence? Did you have any reflections on whether screening for the risk of violence could be considered right or wrong, fair or unfair? If so, how did these considerations influence the development of the training program and the implementation plan?”. Open-ended probes were used to gain depth.

### Analysis

The analysis was performed by the first and last authors and was a combination of conventional and directed content analysis (36). Audio from the interviews was recorded and transcribed verbatim using the AI based transcription instrument Whisper. Transcripts were edited for clarity and checked for accuracy by the first and last authors. We began by reading through all transcripts to get an overview of the entire dataset. Next, we identified content related to any aspect of acceptability, and coded and sorted that content into the seven component constructs according to Sekhons (18) theoretical framework. In the continued analysis for this paper we zoomed in on content relating to the intervention’s ethicality, i.e., “the extent to which the intervention has good fit with an individual’s value system” (18), and what respondents considered to be its ethical strengths and weaknesses. Codes derived from the text were sorted into emergent categories and themes, which are presented below. The thematization was primarily based on unit staff perceptions of ethical aspects of the implementation of routines for structured violence risk screening, while the interviews with the intervention developers were tapped for information and viewpoints relevant to the opinions and concerns expressed by the staff members.

### Ethics

The respondents received written and oral information about the study, and their written consent to participate was collected. Ethics approval was obtained from the Swedish Ethical Review Authority prior to the study (file nr. 2023-05497-01).

## Results

In total, 6 individuals were interviewed: 2 belonging to the development team (one psychologist and one psychiatrist) and 4 staff members in the units in which the intervention was implemented. The staff group consisted of 3 nurses and 1 nursing assistant (1 from the psychiatric unit), aged 33–53 years, whom each estimated that they had been involved in the administration of 7–100 V-RISK-10 screenings.

The analysis resulted in three main themes, see **Table 1**. The first, *A means to promote uniform and fair assessments*, concerns the belief that the routinized use of V-RISK-10 to screen for violence risk promotes uniform and therefore more fair assessments. The second, *Risks of misinterpretation, labelling and negative interaction*, concerns ethical risks that respondents saw in the use of V-RISK-10, tied to how the results of the screening could be misinterpreted by others, resulting in labelling of individuals as inherently dangerous, or having unwanted effects on treatment and staff-patient interaction. The third and final theme, *A balancing of interests*, addresses respondents’ reasoning about the overall ethicality of the intervention in terms of a balance between the interests of staff members and patients respectively, but also between different patient interests.

**Table 1:** Themes of professionals’ ethical acceptability of introducing routine use of Violence risk screening-10 (V-RISK-10).

Themes		
A means to promote uniform and fair assessments	Risks of misinterpretation, labelling and negative interaction	A balancing of interests

### A means to promote uniform and fair assessments

Staff members expressed that a strength associated with the routine use of V-RISK-10 to assess violence risk was that it provided a means to make the management of violence risk more fair and uniform. Based on the belief that violence risk screenings take place and form an integral part of psychiatric care practice regardless if it is done in a formalized and explicit manner or not, the use of V-RISK-10 was understood as a more transparent and objective approach to a problem that was already dealt with on a tacit and subjective case-to-case basis. It was also described as a turn to a more facts-based and less intuitionally informed approach to violence risk screening.

*“An important thing is that no one is, like, shooting from the hip just because [a certain patient] looks scary, but that we assess all patients following the same set of parameters. There is fairness in that, I believe. [-–] Now we have a tool and a scale, something to actually use. Instead of the very subjective thinking [of before].”*

*– Staff 1 (S1)*

Although it was not believed to completely suppress the subjective influence on assessments, the new routine was thus believed to reduce it. Another benefit was how it was thought to increase the predictability of assessments. This was in itself seen as a conflict-reducing feature, as it made it easier for staff to communicate and explain rules and boundaries to patients.

*“[Using V-RISK-10] there is less of a risk that we in the staff are divided up in an “Us” and a “Them”. The good and the bad. It becomes evened out for the patients, helping them to actually handle their situation.”*

*– S3*

### Risks of misinterpretation, labelling, and negative interaction

A particular source of worry surrounding the intervention was how the screening could be misinterpreted, especially by other healthcare providers. Developers and staff all agreed that the aim of the screening was to prevent violence through enhanced preparedness and the opportunity to work with protective factors.

*“We want it to be good. That we get into the know and adapt to reduce the risk of threats or violence, and, you know, put measures in place which will benefit the patient.”*

*– S4*

*“It is more about how we position ourselves, think and work in the staff group. More about general things, rather than in the individual patient encounter. Of course that should not be affected.”*

*– S1*

*“Our intention has been that if you’ve appraised the risk as high, you start by looking at the protective factors.”*

*– Developer 1 (D1)*

At the same time, staff members acknowledged the risk that information about individuals’ results could be misunderstood or misused, especially by professionals from units that had not taken part of the intervention. There was concern that a lack of understanding of what V-RISK-10 is and what it measures – situational and short-term risks – could leave room for emotional knee-jerk reactions from healthcare staff taking part of the documented results, affecting the treatment of patients in unwanted ways. A particular aspect of the instrument believed to be prone to misunderstanding was its limited validity over time, and that it needs to be followed up for results to be practically useful. One staff member saw a risk that, despite this, a single high-risk-result could continue to follow the patient throughout his or her future healthcare contacts, with negative repercussions for the individual.

*“A year back I didn’t know what V-RISK-10 was about, and if I had been told just that there is a high risk for violence and threats, of course I would have been disturbed, especially if it wasn’t something I work with daily. So, there was – and there is – a worry about how other care givers will interpret it. [-–] It is supposed to be treated as a living document, and the assessment is only valid for the episode of care in question.”*

*– S1*

*“The assessment might be from an occasion when the patient was very ill. And then it sticks to them. I have thought about how that could lead to negative things for the patient.”*

*– S4*

In conjunction with this, both the development team and one of the interviewed staff members were concerned that the documentation of violence risk could contribute to an already ongoing stigmatization of psychiatric patients, and to the labelling of patients with high-risk screenings as dangerous, adversely influencing future healthcare contacts.

*“The risk is that … it concerns a fear that the patient is labelled or treated differently than their co-patients. [-–] We have a feeling [ … ] that our patients face quite great trouble in contacts with somatic healthcare, that they can be poorly received or not get the same help as someone without a diagnosis or addiction.”*

*– S1*

*“Unfortunately, the code used for registration [of screening results] is called “Danger assessment”. It should not be called that, but “violence risk”. Because it is not an assessment of a person or an individual but the current risk for violence.”*

*– D2*

Staff members also believed that the use of V-RISK-10 could have adverse effects on staff–patient interactions as well as other aspects of treatment. The admission process could be made more awkward by the need to ask questions about patients’ histories of violence, and there was also a worry that patients could react poorly if they retrospectively found out that a violence risk screening had been conducted on them. A common concern was the risk for discrimination. Several respondents emphasized the need for clear guidelines on what should be the response in case a high violence risk is established. In line with the intentions of the development team, the staff members emphasized that staff should not treat such patients differently than others, except through the enactment of agreed upon measures. Still, they saw a lingering risk that a positive result could affect the reception of the patient and even lead to tangible discriminatory action. It was speculated that it could lead to increased repression, with staff becoming more prone to resort to physical coercion, but also that it could result in different forms of deprivation, e.g., staff attending to patients’ needs less frequently or denying them from taking part of treatment that they would have otherwise benefited from.

*“People may tend to be quicker to take coercive measures because of a fear that there will be threats and violence. And [patients] may not access, or be offered, for instance ASSIP [Attempted Suicide Short Intervention Program] or some other intervention because you think that “no, maybe it is not suitable because of the assessment”.”*

*– S4*

Both among intervention developers and staff members, it was also believed that, without caution, special treatment of patients to manage high violence risk might even make the screening self-fulfilling. It was argued that if patients are attended upon in a way that makes it obvious that they are treated differently, e.g., by being crowded by staff or met by staff members displaying a high-affective behavior, this might in itself provoke violence.

*“It could turn into, you know, “oh now we have done this check, and the person is estimated as really high [risk]”. Then you’re wound up. [ … ] And you enter the patient’s room with a certain attitude. And I think that it might be triggering. [-–] It might be that we ourselves provoke a behavior that wasn’t there to begin with.”*

*– S2*

The developers described that their team had been aware that the prediction of violence risk is fraught with uncertainties, and that the evidence base is not solid regarding the accuracy of screenings. They had therefore avoided to tie the risk screening routine to the use of any repressive measures, or to the discharge procedure. Still, unit staff members saw potential risks associated with inaccurate screenings. One of them argued that the violence risk might be exaggerated in the case of previously unknown first-time patients, when many of the items of the V-RISK-10 cannot be answered with certainty. Another, however, played down the risks tied to exaggeration and instead warned about potential adverse consequences tied to the under-estimation of violence risk.

*“That is actually something that we’ve discussed a little, like, okay we might estimate someone to be at high risk because [there is so much about them that] we don’t know. The only thing we know, maybe, is “severe illness and a bit suspicious”. And because we don’t know much, we estimate the risk as high, just to be safe, but that might not be accurate.”*

*– S4*

*“I don’t think that it is such big of a deal if you mistakenly appraise the risk as high. For example, if you don’t know the patient and put “high”. Because the patient will not be mistreated in any way. [-–] The opposite is probably worse, when you’ve appraised it as low but, actually, it is high. Because then you don’t have the time to make necessary preparations.”*

*– S3*

### A balancing of interests

According to the development team, initially some staff members had outright rejected the idea of routine instrument-based violence risk screening. Meanwhile, at least in retrospect, the staff members interviewed for this study were all positive to the implementation of the intervention, in spite of any ethical problems they perceived. Rather than handling it as a straightforward issue, they assessed the ethical soundness of the intervention in terms of a balance of various interests.

One way in which the dilemma was framed was as a conflict between, on the one hand, patients’ rights to integrity and liberty and, on the other hand, the interests of the staff to protect themselves and others from physical injury or loss of life.

*“I’m marked by having worked here for so many years, subjected to a lot of threats and violence. On many occasions, I have been too tolerant to that. I’ve [ … ] accepted more than what one really should. [ … ] And now I’m thinking, the patient, yes, but [ … ] I am valuable as well. [ … ] I’m positive to this kind of thing.”*

*– S4*

*“There are [ethical risks], absolutely. But it is a difficult problem, I would say. [-–] Of course you must take [risks to the patient] into account, but other things are more important. If it is a matter of preventing physical injury, or someone’s life or health is at stake, you know. And if [the risk of violence] can be minimized based on this, I think that it is important to work with it.”*

*– S2*

The ethicality of the intervention was not only understood in the light of conflicts between the interests of staff and patients, however. It was also described as dependent on the balance between *different* patient interests, in which potential harm in the form of, e.g., discrimination, was weighed against the adverse consequences that violence may have to individual perpetrators themselves. This was mentioned as an important principle guiding the development of the intervention as well.

*“The patient should receive the best possible care. And that will not happen if they are thrown out for being threatening. They miss out on opportunities; they miss out on a chance to get their lives together. [ … ] We should not offer them failure on a plate if we can avoid it.”*

*– S3*

*“Most patients – or almost all patients who act violently [ … ] – it is not to their advantage, but rather a disadvantage. Law enforcement might be called in, and after that you’re labelled as dangerous and might receive poorer quality care and poorer access to care, and so on. So, it lies in the best interest of the patient to avoid getting caught up in violent incidents while they are in psychiatric care.”*

*– D2*

## Discussion

When considering the ethical acceptability of introducing routine violence risk screening as a part of the patient admission process in the studied setting of emergency psychiatric inpatient care, interviewed staff in this study recognized a mix of perceived benefits and risks. The use of V-RISK-10 was considered by the participants to be an ethically acceptable measure, indicating that, despite the presence of perceived ethical risks and conflicts, violence risk screening can be seen as a legitimate measure to enhance safety. The ethical viewpoints of our respondents revolved around the delicate objective of handling risk effectively and respectfully for all involved, where a safe work environment is a prerequisite for providing high quality psychiatric emergency care to patients in need. In the end the perceived ethicality of structured violence risk screening depended on the prioritization of conflicting interests. A classic way to understand the ethical conflict in violence risk assessment is as one where the welfare of the individual is couched against that of the collective (37). Several respondents in this study, however, saw the screening as much as a means to protect patients from the harm they would bring upon themselves through behaving violently.

According to the participants, using an instrument like V-RISK-10 for the screening of violence risk was, if used correctly by trained and experienced staff, considered to render more transparent and uniform results than unstructured clinical assessments. Thus, it was thought to mark an ethically beneficial development away from unstructured, “first generation” (38, p. 650) violence risk assessments. The manner in which it was described as a means to avoid emotionally charged judgements of risk, is particularly relevant in light of how inpatient violence can create a highly emotional and tense ward climate (9).

Potential misinterpretation or misuse of violence risk screening results, meanwhile, formed a major ethical problem in the eyes of our respondents. As in a prior interview study with psychiatric staff, the risk of stigmatization of patients was considered (31). However, whereas the previous study described worries about a stigmatization of psychiatric patients in general, the respondents in our study were more concerned with the possibility that *individual patients* could be labelled as inherently and statically high risk, i.e., dangerous, especially in encounters with healthcare professionals outside the units that participated in the intervention. One perspective on this may be the ethical risks of discrimination in psychiatry, where individuals who are labelled as a risk to others may be at increased risk of coercion (39, 40). This underlines the need for training of healthcare staff in how to interpret documented results of structured screenings for violence risk. The need for training to keep violence risk assessment routines sustainable has been acknowledged before (30). Our findings point towards the necessity of doing so broadly throughout the healthcare organization. Beyond the professionals directly responsible for the conduction of violence risk screenings, staff in other units throughout the same organization need to maintain a sufficient level of violence risk assessment literacy as well, for the screening practice to seem ethical. It also highlights the significance of guidelines ensuring that screening results are documented and communicated in a way that facilitates their interpretation by other caregivers. Intertwined with concerns regarding information use were worries that professionals’ reactions to screening results could be arbitrary, and that patients found high-at-risk might face discrimination or maltreatment. A need for robust guidelines was expressed, to promote clarity on how to manage the risk that has been assessed, i.e., measures tied to the different outcomes.

That violence risk assessment procedures are sometimes believed to be awkward or disruptive to the interactions between healthcare staff and patients is a concern that has been reported before (30, 41). In the intervention studied, the screening could be carried out completely without the knowledge of the patient. This created several concerns. There was a belief that the need for information about past violent events introduced a potentially awkward step into the admission procedure. Also, since patients have access to their medical records, and could thus read about the screening and its results afterwards, leaving staff wondering how patients would react. One way to decrease the risk for adverse emotional reactions to such revelations – and at the same time also dampen the risk for situations arising during the conduction of the screening – would be to involve the patient more actively in the screening process, for example by including elements based on self-perception (42), which may be effective (43) and in line with the concept of participation in healthcare (44).

A previously known challenge perceived by psychiatric care staff in the completion of risk assessments is how they are often shaped by incomplete information (30, 31). This was acknowledged by respondents in the current study as well. Prior research has indicated that lack of information at the time of admission is associated with increased risk of violence during admission (45). Overall, however, problems tied to poor accuracy – an issue that have been subject to considerable debate (23–26) – did not appear to be a great problem to the respondents. This might be because V-RISK-10 is an instrument for structured professional judgment rather than an actuarial tool (34), resulting in less emphasis on prediction. However, even though it does not eliminate all issues tied to resource management, it might also be a result of the strong emphasis on prevention in its current implementation, and the focus on preventive risk management built around non-coercive measures and protective factors.

### Methodological reflections, strengths, and limitations

The low number of study participants is a limitation to this study. We would have wished to include more informants in our study, but despite efforts, recruitment proved difficult. All individuals fulfilling inclusion criteria were informed about the study, and invited to participate, on several occasions. Possible reasons for the reluctance to participate include a high workload among unit staff, and fatigue associated with continuous development work in the units. However, it must also be considered that staff members who were critical to the intervention might have been reluctant to volunteer. It is a strength of the study that those who did volunteer to be interviewed contributed generously with different perspectives which point towards our results being of value. However, despite any objections they had, all of them displayed a positive attitude to the intervention as a whole. It is possible that the inclusion of a greater number of respondents would have resulted in a wider representation of viewpoints, including more negative views of the ethicality of structural violence risk screening. Possibly, staff members with more negative views could also have different perceptions on, for instance, which interests that are at stake, or how they should ideally be balanced. These aspects makes it important to read the results with a certain caution, and it calls for further studies of this aspect of violence risk screening. However, even though more participants with a potentially wider representation of views on ethicality could have made for a broader and perhaps more nuanced analysis, we still believe that the analysis as it stands highlights central aspects of how psychiatric staff may perceive the ethicality of violence risk assessment. This study concerned the ethicality of violence risk screening as perceived by intervention developers and staff members in acute psychiatry settings. It says nothing about either its effectiveness or attitudes towards such interventions among patients. Future research could look at screening results, outcomes, and patient experiences.

## Conclusion

The emergency psychiatric staff members interviewed in this study were all positive to the use of V-RISK-10 to screen patients for violence risk as a part of admission to their units, despite voicing concerns regarding the ethicality of doing so, and the ethical risks involved. The overall ethical acceptability of the intervention was decided in a process of complex balancing of interests, weighing against each other individual and collective interests, as well as conflicting interests of the individual patient. However, the problems staff members recognized suggest that the ethical acceptability of structured violence risk screenings can be enhanced both by a wide dissemination throughout the organization of knowledge on how to interpret screening results, and by the provision of clear guidelines on how they should shape the management of violence risk.

## Data Availability

The datasets presented in this article are not readily available because of sensitive and confidential information. Requests to access the datasets should be directed to lars.garpenhag@med.lu.se.
